# Comparison of the efficacy of altrenogest versus transdermal progestin patches on estrus synchronization and ovulation in mares

**DOI:** 10.14202/vetworld.2023.1667-1672

**Published:** 2023-08-19

**Authors:** Jatuporn Kajaysri, Supphathat Wutthiwitthayaphong

**Affiliations:** 1 Chulabhorn Royal Academy, Bangkok, 10210, Thailand; 2 Clinic for Obstetrics and Gynecology Andrology and Artificial Insemination of Domestic Animals, Faculty of Veterinary Medicine, Mahanakorn University of Technology, Bangkok, 10530, Thailand

**Keywords:** altrenogest, estrus synchronization, mare, progestin, transdermal patch

## Abstract

**Background and Aim:**

The adhesive progestin patch was investigated for estrus synchronization in mares because this method is convenient and safe in other species. This study aimed to compare the efficacy of a transdermal progestin patch versus oral altrenogest on estrus synchronization, preovulatory follicle development, and ovulation.

**Materials and Methods:**

Twenty-four broodmares were randomly divided into two groups. In Group 1, mares (n = 12) were fed 0.044 mg/kg altrenogest daily for 14 days. Group 2 (n = 12) mares were treated with adhesive transdermal progestin patches for 14 days. Mares were observed for estrus daily, from day 3 until day 7, after hormone withdrawal. Follicular development was determined by ultrasonography. The preovulatory follicle size and ovulation time after hormone treatments and ovulation rate were determined for both groups. Plasma progesterone levels were measured during the experimental periods and 2 days after hormone termination in both groups.

**Results:**

The results revealed that the transdermal progestin patch efficiently controlled follicular growth and estrus synchronization in mares. The percentage of mares exhibiting estrus was similar in the altrenogest (100.00%) and transdermal patch (91.67%) groups. Ovulation rates were equivalent with either altrenogest or progestin patch protocols (91.67% [11/12] vs. 83.33% [10/12]). In addition, the preovulatory follicle size was similar in mares treated with altrenogest and progestin patches.

**Conclusion:**

This study revealed that the transdermal progestin patch provides effective estrus synchronization and ovulation, similar to altrenogest treatment. However, the transdermal patch was more convenient with a shorter predictable ovulation time after estrus synchronization and should be considered as an alternative method for mares.

## Introduction

Estrus synchronization is a beneficial tool for breeders with many mares. This method facilitates the prediction of the onset of estrus, reduces the need for estrus detection, and saves both time and money [1]. The profitability of horse breeding as a successful agricultural enterprise will increase if the breeders can lower the investigation costs [2]. However, synchronization of estrus and ovulation in mares is difficult because the follicular phase is wide and variable (2–18 days), primarily due to environmental influences, such as day length [3]. Intravaginal progesterone-releasing devices efficiently synchronize estrus in mares. However, vaginitis with mild purulent discharge was observed in some mares after the insertion of such devices [4]. Such reactions may influence practitioners to seek other optimal methods for estrus synchronization in mares. An oral progestogen, such as altrenogest, is commonly used for estrus and ovulatory synchronization in broodmares [4]. Altrenogest is a 17-alpha-allyl derivative of a trenbolone agent that maintains mares’ reproductive cyclicities [5]. It is typically added to feed as a powder or liquid daily for 10–14 days [6]. This long-term administration of altrenogest functions as a synthetic progesterone, artificially prolonging the luteal phase until natural corpus luteum (CL) regression, while allowing the normally growing follicle to continue growing. Altrenogest suppresses luteinizing hormone (LH) secretion to prevent ovulation [7, 8], and the dominant or maturation follicle subsequently ovulates within 10 days after the termination of treatment [6]. The interval from altrenogest termination to ovulation may vary in mares. Using progesterone followed by a luteolytic agent, such as prostaglandin F_2α_ (PGF_2α_), at the time of the progesterone withdrawal may result in regression of the remaining CL after progesterone treatment [9], which may lead to a shorter and more consistent time interval between altrenogest withdrawal and ovulation. However, using altrenogest in horses is impractical for treating many mares daily for a long period (10–14 days). If mares are housed in groups, it is also more difficult to control the consumption of the correct dosage of the consumed hormone. In addition, current research on using altrenogest in mares after rectal, vaginal, or injectable administration indicates that the effectiveness of altrenogest is adequate to suppress estrus behavior in mares [10–12]. Even more recently, there was a report on using a progesterone-releasing intravaginal device to control estrus and ovulation in mares [13].

Therefore, it would be beneficial to have an alternative method that is easy to use and effective for estrus and ovulatory synchronization, while not interfering with the fertility of mares. Recently, the combined hormonal contraceptive transdermal patch has been extensively used in women. This commercially available medication contains progestin norelgestromin and estrogen ethinyl estradiol (Evra^®^ or Ortho Evra^®^, Ortho-McNeil-Janssen Pharmaceuticals Inc., New Jersey, USA) [14]. A single adhesive patch of 20 cm^2^ size delivers a daily dose of 150 μg norelgestromin and 20 μg ethinyl estradiol for 7 days, which is sufficient to inhibit ovulation for 9 days in women [14]. Kajaysri *et al*. [15] compared the progestin transdermal patch and PGF_2α_ administration versus the controlled internal drug-release device plus PGF_2α_ protocol for estrus synchronization in beef cattle. It was found that the progestin patch with PGF_2α_ was as effective as the controlled internal drug-release device with PGF_2α_ protocol at synchronizing estrus. In contrast to the internal drug-release device, the progestin patch protocol did not cause bacterial infection or irritation in the reproductive tract of cows, because this method did not directly administer treatment to the reproductive tract. Considering these results, the progestin patch with PGF_2α_ treatment may provide a practical protocol for estrus synchronization with no detrimental effect on reproductive organs in mares.

This study aimed to compare the efficacy of oral progestogen (altrenogest) and transdermal progestin patch protocols for estrus synchronization and ovulation in mares.

## Materials and Methods

### Ethical approval

The study was approved by the Animal Ethics Committee of Faculty of Veterinary Medicine, Mahanakorn University of Technology, Bangkok, Thailand (approval no. ACUC-MUT-2022-005).

### Study period and location

The study was conducted during the natural breeding season (March–July 2018) with outdoor temperatures ranging from 25°C to 40°C and an average relative humidity of 70.5%. The study was conducted at a Stud farm in Nakhon Ratchasima province, Thailand.

### Experimental animals

Twenty-four healthy and normally cycling mares of the thoroughbred breed at 5–12 years of age were randomly selected. The parity of these pluriparous broodmares ranged from 1 to 4. Their average body weight was 420 ± 76 kg (mean ± standard deviation [SD]) and their body condition score was about 4–6 out of 9 according to the scale of Henneke *et al*. [16]. Before the study, each mare was monitored by transrectal ultrasonography (7.5 MHz, B-mode, linear-array transducer scanner; Sonoscape A6^®^, SonoScape Co., Ltd., Shenzhen, China) to determine ovarian activities and any reproductive problems. All mares in this study exhibited the estrous cycle with the presence of follicles and or CL and were free from any anatomical or reproductive disorders. The mares were housed in a free stall barn and fed daily with roughage (fresh and dry Pangola grass) and concentrates containing 14% of crude protein and had access to clean water *ad libitum*. They could exit the stall barn in the morning to walk freely in a grass field and return in the evening. The mares were dewormed six times a year and routinely vaccinated for disease prevention following the standard vaccination program provided by the American Association of Equine Practitioner Infectious Disease Committee (2008).

### Experimental design

**Figure-1:**
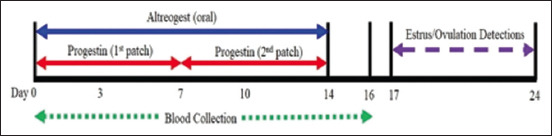
Diagram of altrenogest or progestin patch hormonal treatments for 14 days followed by estrus and ovulation detection and blood collection from mares. Blood collections were done once daily from day 0 until day 16. Day 0 was the starting day for both protocols.

**Figure-2:**
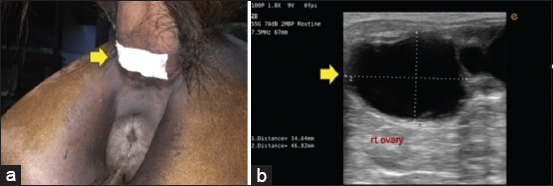
(a) Adhesive area of the transdermal progestin patch (arrow) at the proximal part of the tail. (b) Detection of dominant follicular development (arrow) from an average diameter of approximately 35 mm until the largest preovulatory follicle before ovulation in mares.

Twenty-four broodmares were randomly assigned to two equal groups. Group 1: 12 mares were fed with altrenogest (Regumate^®^; 0.4% weight/volume, MSD Animal Health, New Jersey, The United States) at 0.044 mg/kg of body weight once a day for 14 days. Group 2: the remaining 12 mares were treated with adhesive transdermal progestin patches (6.00 mg norelgestromin and 0.75 mg ethinyl estradiol; Ortho Evra^®^, Ortho-McNeil Pharmaceuticals, Raritan, New Jersey, USA) for 14 days, placed on the skin at the ventral side of the proximal part of the tail. Two adhesive patches were used for Group 2 animals, with one patch used for 7 days then removed and immediately replaced with a second patch for another 7 days of treatment, resulting in a total treatment of 14 days. The adhesive area was chosen because the skin had less hair, was soft and thin, and the patch could be easily pasted. Finally, patches were covered with adhesive tape to protect them from urine and feces during treatment. The examination of any complications of altrenogest and progestin patch treatments included examination for the presence of obvious allergic reactions as well as the skin at the adhesive area immediately after the withdrawal of altrenogest or patches. A diagram of the experimental design is shown in Figure-1. The adhesive area of the transdermal progestin patch at the proximal part of the tail is shown in Figure-2.

### Determination of plasma progesterone levels

Daily blood samples (5 mL/mare; in heparin anticoagulant tubes) were collected from all mares of the two groups for 17 days to measure the plasma progesterone levels. The sample collection was started on day 0, which was the day before hormonal treatment (start of both treatment groups), continued on day 1 to day 14 (days during altrenogest or patch treatment), and through days 15 and 16 (1 and 2 days, respectively, after altrenogest or progestin patch withdrawal). All the blood samples were stored on ice and transported to a laboratory at the Research and Development Centre for Livestock Production Technology, Faculty of Veterinary Science, Chulalongkorn University, Bangkok, Thailand. Blood samples were centrifuged at 3000× *g* for 5 min to collect plasma samples, which were stored at −70°C until the measurement of progesterone levels using a radioimmunoassay. The plasma (50 μL in duplicate) sample progesterone levels were measured using a direct radioimmunoassay and Danazol^®^ Reagent (Sigma-Aldrich, CAS No. 17230-88-5, Missouri, USA) as a displacing agent. In addition, plasma samples were tested in the three pools (low, medium, and high) of the progesterone standard. The intra- and inter-assay coefficients of variation for the three pools were 8.03%, 9.95%, and 8.50% (intra), and 19.00%, 18.80%, and 11.73% (inter), respectively. The sensitivity threshold of the test was <0.01 ng/mL.

### Estrus and ovulation detection

After the termination of hormone treatment in mares, the estrus response in all mares of both groups was examined using transrectal ultrasonography. The detection of uterine endometrium edema (Grade 1 from 4) and a dominant follicle (diameter of approximately 35 mm) in both ovaries indicated a mare in estrus according to studies of Canisso *et al*. [4] and Bruemmer *et al*. [17]. For the entire experiment, examination for estrus detections, their fertility, follicular development, and ovulation was performed by only one experienced veterinarian once daily using transrectal ultrasonography of both ovaries of mares in both groups for 8 days after hormone withdrawal, starting 3 days after hormone termination (day 17–24). The numbers of mares in estrus in each experimental group were recorded. The maximal diameters of the largest or expected dominant follicles were recorded daily using ultrasonography. The presence of a dominant follicle was examined until ovulation. The sizes of preovulatory follicles in mares of both groups were recorded. Ovulation was defined by the absence of a dominant follicle and the presence of a subsequent CL at the same site in the ovary. Mares that ovulated in both groups were recorded to determine the ovulation rate of each group. The approximate intervals (days) to ovulation after the hormone withdrawals were recorded for both groups.

### Statistical analysis

The data obtained for follicle development were descriptively expressed. The estrus, complication, and ovulation rates were statistically assessed using the χ^2^ test with or without Yates correction. An independent t-test was used to compare the means (±SD) of preovulatory follicle sizes and the number of days to ovulation after hormone treatments. Furthermore, the plasma progesterone concentrations in both groups were analyzed using a linear mixed model procedure. A probability of p ≤ 0.05 was considered significant. All statistical analyses were performed by Program R (R Foundation for Statistical Computing, Vienna, Austria).

## Results

A total of 12 mares (12/12, 100%) showed an estrus response (uterine endometrium edema and dominant follicles) about 3–5 days after altrenogest withdrawal. In comparison, the estrus response was observed in 11 mares (11/12, 91.67%) 3–5 days after the removal of transdermal progestin patch treatment.

The estrus rates of Groups 1 and 2 were not significantly different. The recruited dominant follicles of mares in estrus in both groups were observed until ovulation or 10 days after altrenogest or progestin withdrawal. The ovulation rates (ovulation numbers) of mares in estrus in Groups 1 and 2 were 91.67% (11/12) and 83.33% (10/12), respectively, and were not significantly different. The average maximal diameters (mean ± SD) of the largest preovulatory follicles about 1 day before ovulation in Groups 1 and 2 were 41.56 ± 0.74 mm (n = 11) versus 40.49 ± 0.84 mm (n = 10), respectively, and were not significantly different.

The patterns of dominant follicular development in terms of average diameter, from approximately 35 mm until the largest preovulatory follicle before ovulation, of mares in estrus are presented in Figure-2. The means (±SD) of the intervals (days) to ovulation after altrenogest or progestin patch withdrawal for Groups 1 and 2 were 9.27 ± 0.79 days (n = 11) and 7.40 ± 0.97 days (n = 10), respectively. The time interval between the day of progestin removal and ovulation was significantly shorter for the progestin patch group compared with the altrenogest group (p ≤ 0.05).

**Figure-3:**
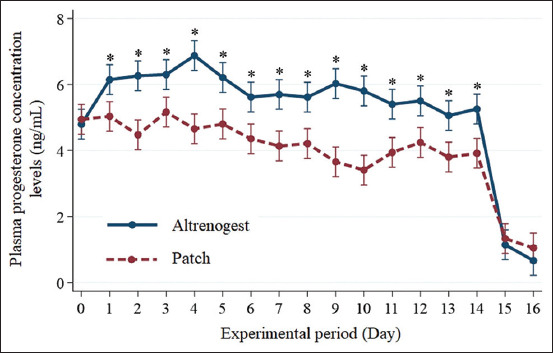
The plasma progesterone concentrations (mean ± standard derivation) of mares from the day of starting the protocol (day 0), then during altrenogest or patch treatments from day 1 to day 14, and 2 days after termination of hormone treatments (day 15–day 16). *Within day 1–day 14 the means were significantly different (p ≤ 0.05) between the treatment groups.

After hormone treatments, the mares in the altrenogest and progestin patch treatment groups did not have any complications, such as purulent discharge or any clinical signs related to pyometra or vaginal infection, and their obvious allergic reactions and inflammation or irritation of skin on treatment areas continued after patch removal. For mares in Group 1, the mean (±SD) plasma progesterone concentration was 6.15 ± 1.30 ng/mL on day 1, then reached the highest level at 6.88 ± 0.92 ng/mL on day 4, followed by 6.21 ± 1.03 ng/mL on day 5 and quite stable high levels until 5.26 ± 0.85 ng/mL on day 14 of hormone treatment. The progesterone levels decreased to 1.15 ± 0.35 and 0.67 ± 0.21 ng/mL on day 15 and 16 (1 and 2 days after altrenogest withdrawal, respectively). For mares in Group 2, the mean (±SD) plasma progesterone concentration was 5.03 ± 0.58 ng/mL on day 1 and then achieved the highest level of 5.17 ± 0.52 ng/mL on day 3, then 4.66 ± 0.69 ng/mL on day 4, and quite stable at moderate levels until 3.92 ± 0.65 ng/mL on day 14 of patch treatment. One–2 days after patch removal, progesterone levels decreased to 1.34 ± 0.90 and 1.05 ± 1.19 ng/mL, respectively. The mean plasma progesterone concentrations were significantly different for mares in Group 1 versus Group 2 during hormone treatments (from day 1 until day 14), as shown in Figure-3. Furthermore, the effects of changing plasma progesterone concentration levels over time in Group 1 were significantly different from day 1 (p < 0.05), whereas Group 2 was significantly different from day 6 (p < 0.05).

## Discussion

The methods of synchronization in this study were excellently successful due to the mares in both groups that had high estrus and ovulation rates. The reason might be that this study was conducted in a season with long days, when photoperiod influenced the endogenous reproductive hormones and enhanced ovarian activity along with synchronization. According to a previous report by Crabtree [18], the reproductive cycle of the horse species is endogenously cyclical, controlled by photoperiod, and synchronizes with the spring. In addition, the start of estrus after synchronization was identical in both groups and according to the onset of mare estrus following synchronization by PGF_2α_ [19].

The results showed that altrenogest (synthetic progestin) was effective for estrus synchronization and ovulation in broodmares, which is consistent with previous studies by Bergfelt *et al*. [3], Hodgson *et al*. [5] and Loy *et al*. [11]. On the other hand, exogenous progesterone hormone therapy has been linked to an elevated incidence of pyometra disease in female dogs and cats. Cystic endometrial hyperplasia, one of the endometrial abnormalities, is considered to be associated with pyometra in dogs and cats due to the progesterone hormone’s significant role in predisposing variables [20, 21]. Therefore, to ensure the safety of the altrenogest and progestin patch treatments, the reproductive organs were concerned to be monitored in this study. Therefore, altrenogest provides an available method to synchronize estrus and ovulation without disturbance of fertility in mares. However, the use of altrenogest is impractical for the long-term treatment (10–14 days) of many mares. This study demonstrated for the first time an alternative method using the transdermal progestin patch for estrus synchronization in mares. The estrus and ovulation rates of progestin patch group were equivalent to those of the altrenogest group.

In addition, the use of progestin patches did not adversely affect reproductive organs nor lead to inflammation of skin at the adhesive areas. The exogenous progestin patch treatment and traditional oral altrenogest protocol had comparable efficacy at inducing estrus in mares, but the patch method was simpler than oral altrenogest, which had to be fed to mares daily for 14 days. Moreover, the patch had no complications compared with intravaginal progesterone-releasing devices [4]. Our study showed that the transdermal progestin patch synchronized estrus in mares with an efficacy similar to that in a previous study by Kajaysri *et al*. [15] using the progestin patch in cattle. In this experiment, the progestin (Norelgestromin) in the transdermal patch was efficiently transdermally absorbed through the skin, consistent with previous research by Burkman [14]. Therefore, it is likely that norelgestromin from the patch functioned like natural progesterone to suppress follicular stimulating hormone secretion and follicular growth, as well as LH release to inhibit ovulation throughout the patch administration of 14 days. With the CL regressed by natural PGF_2α_, mares treated with altrenogest exhibited estrus after progestin removal, consistent with the reported effects of altrenogest [6–8]. However, altrenogest may not completely suppress follicular stimulating hormone secretion and all follicle growth [7]. The follicular wave also continued during transdermal patch treatment, reflected by the existing follicles that slowly grew to medium size during patch treatment of mares. Such growth is similar to that observed with oral progestogen in the form of altrenogest, which influences follicle-stimulating hormone and follicles in mares [6]. With the growing follicle further developing during progestin patch treatment, following the withdrawal of progestin, the growing follicle would be able to develop into a dominant follicle, and the surge of LH secretion would stimulate its ovulation at a predictable time, similar to the altrenogest protocol [6]. The present study found that the sizes of preovulatory follicles were not different between the altrenogest and progestin patch groups, suggesting that both hormone treatment protocols had a similar efficiency in affecting follicular growth in mares. In addition, the preovulatory follicle size of mares treated with altrenogest in this study was similar to that of a previous study by Canisso *et al*. [4] and James *et al*. [22]. The present study also found that the number of days from hormone withdrawal until ovulation for mares treated with altrenogest was similar to that found in a previous report by Norman and Larsen [6].

However, the mean number of days to ovulation after withdrawal of progestin patch treatment was shorter than for the mares in the altrenogest group. One reason may be the lower plasma progesterone concentrations for mares in the progestin patch group (from day 1 until day 14 of treatment) compared with the altrenogest administration group. Altrenogest promoted higher plasma progesterone concentrations during treatment, thereby postponing the ovulation time in mares, consistent with the efficiency of oral progestin reported in previous studies by Canisso *et al*. [4] and Bruemmer *et al*. [17]. The present findings also support research by Wiepz *et al*. [23], showing that altrenogest administration suppressed the preovulatory surge of LH and delayed ovulation in mares. The time for ovulation after altrenogest treatment in this study was similar to a previous report by Norman and Larsen [6]. The transdermal patch also contained ethinyl estradiol, which may affect follicle growth. The combination of progesterone and estrogen at the beginning of estrus synchronization has been shown to suppress follicle growth and cause the appearance of a new follicular wave [8, 24].

The results analysis of this study showed that the plasma progesterone level pattern following oral altrenogest treatment was similar to that of a prior study [11]. As a result, oral altrenogest can influence estrus behavior suppression and estrus synchronization by supplying and maintaining a high amount of plasma progesterone. An important finding in the present study was the differences in plasma progesterone levels during and after progesterone treatments in both groups. The significantly lower plasma progesterone levels in mares treated with transdermal patches compared with altrenogest may reflect a lower amount of progestin in the patches (6.0 mg norelgestromin), and the skin layer presenting a barrier for hormone absorption, as proposed in a previous study by Kajaysri *et al*. [15] using progestin patches in cattle. However, the lower plasma progesterone levels from the patch still effectively synchronized mares, similar to the traditional oral altrenogest method. A comparison of synchronization efficiency indicated that norelgestromin provided hormone potency similar to altrenogest. In addition, the effects of changing plasma progesterone concentration levels over time in mares with patch treatments differed from day 6, while those in mares with oral altrenogest treatments differed from day 1. That is, it may affect the patch’s slow release of norelgestromin day by day to the blood vessel through the skin and dermis, allowing it to take longer for accumulation into a higher level of plasma progesterone concentration.

On the other hand, oral altrenogest could have a fast effect and a higher plasma progesterone concentration because it might be absorbed from the intestine to the bloodstream as well. However, using progestin patches during 14 days for estrus synchronization in mares was not the most convenient method, because the adhesive patches were replaced after 7 days. Thus, the use of progestin patches in short protocols (for 7 days) may be more convenient, but PGF_2*α*_ should be used immediately after patch removal so that the timing of estrus and ovulation rates in mares are similar to those in a previous report by Ginther *et al*. [9] using progesterone with PGF_2*α*_ protocol. The present study suggests that the progestin absorbed from dermal patch could effectively synchronize estrus and ovulation without affecting fertility. However, further studies should inseminate mares without initial detection of estrus and then after estrus and ovulatory synchronizations by the progestin patch and evaluate pregnancy rates using this treatment protocol.

Overall, this study revealed that the progestin patch protocol maintained the ability to recruit the dominant follicle. The transdermal progestin patch had a similar efficiency compared with the oral altrenogest protocol in terms of estrus and ovulation synchronizations. Even though there are a number of altrenogest products available now, including oral, injectable, transrectal, and transvaginal administrations, they are still impracticable to use for synchronizations in mares. Furthermore, the FDA [25] is aware that the altrenogest hormone poses potential risks for humans who come into contact with contaminated equipment, barn surfaces, or treated animals because the hormone is able to absorb through intact skin. In addition, this novel progestin patch is a human product, and its approach in mares demonstrated no tissue reactivity or reproductive disturbance, which should be highly practical for horse breeding.

## Conclusion

This study revealed that altrenogest and the transdermal progestin patch could efficiently synchronize estrus and control follicle development until ovulation. Conversely, the transdermal patch was more practical for mares because it had quicker, more predictable ovulation timing after estrus synchronization. Further studies are recommended to evaluate the use of transdermal progestin patches in timed artificial insemination and embryo transfer protocols as an alternative method to enhance reproductive performance in mares.

## Authors’ Contributions

Both authors participated in the experimental design and conducted the experiment. JK: Conducted a comprehensive literature search, analyzed the collected data, drafted, revised, and proofread the manuscript. SW: Collected the data. Both authors have read, reviewed, and approved the final manuscript.
